# Analysis of the content consumed by internet addicted adolescents of central siberia: Gender and age differences

**DOI:** 10.1192/j.eurpsy.2021.1544

**Published:** 2021-08-13

**Authors:** N. Semenova, S. Tereshchenko, L. Evert L, Y. Kostyuchenko, M. Shubina

**Affiliations:** Scientific Research Institute For Medical Problems Of The North, Federal Budgetary Scientific Institution «Federal Research Centre «Krasnoyarsk Scientific Centre of Siberian Division of Russian Academy of Sciences», Krasnoyarsk, Russian Federation

**Keywords:** Internet, Addiction, Siberia, adolescents

## Abstract

**Introduction:**

Teenagers’ Internet addiction can be supported by a wide variety of Internet content.

**Objectives:**

To study the structure of the content consumed by Siberian adolescents with Internet addiction.

**Methods:**

200 (69 boys and 131 girls) Internet addicted adolescents aged 11-18 years and living in the urban area of Central Siberia (Krasnoyarsk) were surveyed. Content consumption was studied using Game Addiction Scale for Adolescents and The Social Media Disorder Scale.

**Results:**

19.0% of adolescents were addicted to Internet games, 22.5% of adolescents were addicted to social media. A combination of both types of addictions was found in 23.5% of adolescents. Other types of content addiction was found in 35% of adolescents. Boys prefer Internet games (62.3% of boys vs. 32.1% of girls), while girls prefer communication on social media (55.0% of girls vs. 29.0% of boys), p <0.001. Combined addiction is observed equally in both sexes (23.2% and 23.7% respectively). For older adolescents, there is observed a decrease in the interest to Internet games (from 48.4% at 11-14 y.o. to 37.6% at 15-18 y.o.) and to social media (from 49.5% to 43.1%). At the same time, interest to other types of content is growing (from 27.5% to 41.3%).

**Conclusions:**

Boys with internet addiction are more likely to be addicted to internet games, while girls are more likely to get engaged in social media. Older adolescents show a decrease in the interest both to Internet games and social media, while their interest to other types of content increases. The study was funded by RFBR project № 18-29-2203218.

**Conflict of interest:**

The study was funded by RFBR project № 18-29-2203218.

